# A Novel Treatment of Fascial Pain With Glucopuncture: Three Clinical Cases

**DOI:** 10.7759/cureus.59544

**Published:** 2024-05-02

**Authors:** King Hei Stanley Lam, Jan Kersschot, Teinny Suryadi

**Affiliations:** 1 Clinical Research, The Hong Kong Institute of Musculoskeletal Medicine, Kowloon, HKG; 2 Faculty of Medicine, The Chinese University of Hong Kong, New Territories, HKG; 3 Faculty of Medicine, The University of Hong Kong, Hong Kong, HKG; 4 Family Medicine, Private Practice, Antwerp, BEL; 5 Physical Medicine and Rehabilitation, Hermina Podomoro Hospital, Jakarta, IDN

**Keywords:** chronic neck pain, arm pain, buttock pain, chronic low back pain (clbp), groin pain, neuropathic pain, musculoskeletal pain, biotensegrity, glucopuncture, fascia

## Abstract

The fascial system (FS) represents a sophisticated and intricate network within the human body, comprising both superficial and deep fascial layers. Disruptions or dysfunctions within this system have been implicated in a variety of musculoskeletal (MSK) disorders and pain syndromes. Specifically, fascial tightness has been associated with diminished range of motion and localized pain. Glucopuncture, a novel therapeutic approach, involves the administration of 5% dextrose injections directly into the fascial layers, such as the superficial fascia, to address these issues. This article presents a case series involving three patients who underwent palpation/ landmark-guided glucopuncture for the treatment of superficial fascial dysfunction. The first case involves a 45-year-old male with a nine-month history of left groin pain, who experienced significant pain relief following glucopuncture below the inguinal ligament, with complete resolution of symptoms within four weeks. The second case describes a 36-year-old female suffering from left arm and neck pain for two years, who reported gradual pain alleviation over six weeks after receiving multiple injections in the fasciae of the neck, scapula, and lateral aspect of the triceps muscle. The final case involves a 67-year-old female with a six-month history of low back and buttock pain, who showed improvement after four weeks following multiple injections in the fasciae of the low back, lumbar region, and buttocks. These cases highlight the potential of palpation-guided glucopuncture as a simple, cost-effective method for modulating regional pain caused by superficial fascial dysfunction. However, further research is necessary to fully ascertain the efficacy and safety of glucopuncture for treating fascial dysfunction.

## Introduction

The fascial system (FS) is an important but often neglected anatomical part of the human body. It is a complex network of connective tissue that runs throughout the body, similar to a three-dimensional spider web. Composed of collagen and elastin fibers, it weaves a web-like matrix surrounding every muscle, nerve, blood vessel, and organ. This intricate network provides structural support, facilitates movement, and plays a crucial role in overall health and well-being. Recent research indicates that fascia acts as a dynamic, flexible single unit that is capable of adapting its biomechanical properties. Fascia also contains many sensory receptors responsible for musculoskeletal (MSK) pain that is inconsistent with medical diagnoses based on magnetic resonance imaging (MRI) or ultrasound. Managing vague MSK pain syndromes can be challenging for both physicians and patients. Glucopuncture has been suggested as a new tool to address such atypical pain patterns by administering multiple injections of low concentrations of glucose or dextrose into the regional fascia. However, clinical correlation does not always indicate true causation. To further elucidate the significance of this correlation, hard data must be obtained. Therefore, additional fundamental and clinical research is urgently warranted to confirm glucopuncture as an appropriate treatment for atypical pain patterns.

The MSK system comprises the myofascial and skeletal systems. The myofascial system comprises both contractile muscle (myo-) and connective tissue (-fascial) [1]. While the skeletal muscles play a basic role in both movement and posture, muscles and bones cannot function on their own. Ligaments, tendons, and fascia are required for skeletal muscles to function properly and facilitate posture and body movement [2]. Ligaments typically connect bone to bone, and tendons connect muscle to bone, while the FS plays a much more complex role [3]. The FS connects the dermis, muscles, and even internal organs of the entire body into one complex network. Especially over the last decade, research has supported the notion that the FS is an important but often neglected anatomical part of the MSK system. The FS acts as a “parallel system,” which is present throughout the body [4]. While it has biomechanical properties, the FS is also an important nociceptive system. Thus, when managing patients with MSK dysfunction, such as reduced range of motion (ROM) or regional pain, the fascia may be an important component to address.

The participation of fascia in myofascial pain syndrome has often been neglected [5]. In contrast to its perceived focus, the textbook by Travell and Simons about myofascial pain syndrome (1992) focused on muscle rather than fascia [6]. Even today, many physicians have not been trained to consider the importance of the FS when treating patients with MSK pain or dysfunction [5]. The FS forms a complex network of connective tissue that runs throughout the body like a three-dimensional structure. It is made up of collagen and elastin fibers, forming a web-like matrix that surrounds every muscle, nerve, blood vessel, and organ in the body. The FS represents about 20% of human body weight. On a small scale, the FS provides structure, protection, and support to muscles, organs, bones, and a variety of fragile internal structures. On a large scale, it forms a supportive structure that is crucial to maintain the body's shape and posture. Fascial connective tissues consist of two components, cells and the extracellular matrix (ECM). Most of the cells in these tissues are fibroblasts, which function as construction and maintenance workers for the surrounding matrix and account for only 5% of the total fascial volume. The ECM consists of ground substance and fibers. Most of the fibers are collagen fibers, except for a few elastin fibers. Although the fascial tissues are primarily soft and pliable, some, such as the iliotibial band, plantar fascia, and lumbodorsal fascia, are rather rigid and strong [7]. Fasciae are organized in layers, and fascial mobility plays a major role in optimal MSK movement, especially along shear planes separating muscles, which is especially important for athletes. Fascia itself may also have some active contractility [7]. In certain areas, it is also connected to the lymphatic system [8]. Dysfunction in the FS has been postulated to be closely associated with the development of a variety of MSK disorders and pain syndromes. This manuscript reports three clinical cases of chronic MSK disorders and pain syndromes treated with glucopuncture of superficial fasciae.

## Case presentation

Clinical case 1

A 45-year-old male experienced left groin pain for nine months. The pain had a gradual onset and was described as a progressive ache. He did not recall a specific injury that triggered the pain. While the pain did not hinder his ability to work as a copywriter, it was bothersome during periods of rest. Although it did not disrupt his sleep, prolonged sitting exacerbated the pain, which he rated as a 6 out of 10 on a numerical rating scale (NRS). While walking, the pain was a 4 out of 10 on the NRS.

Despite experiencing pain for nine months, the patient did not seek formal medical attention. This was due to two main factors: his busy schedule and a preconception, formed from previous reading, that pain intervention might involve steroid injections, which he believed could lead to long-term soft tissue, cartilage, and bone damage. He attempted self-stretching and massage at home, which occasionally provided some relief. However, the pain persisted.

**Figure 1:**
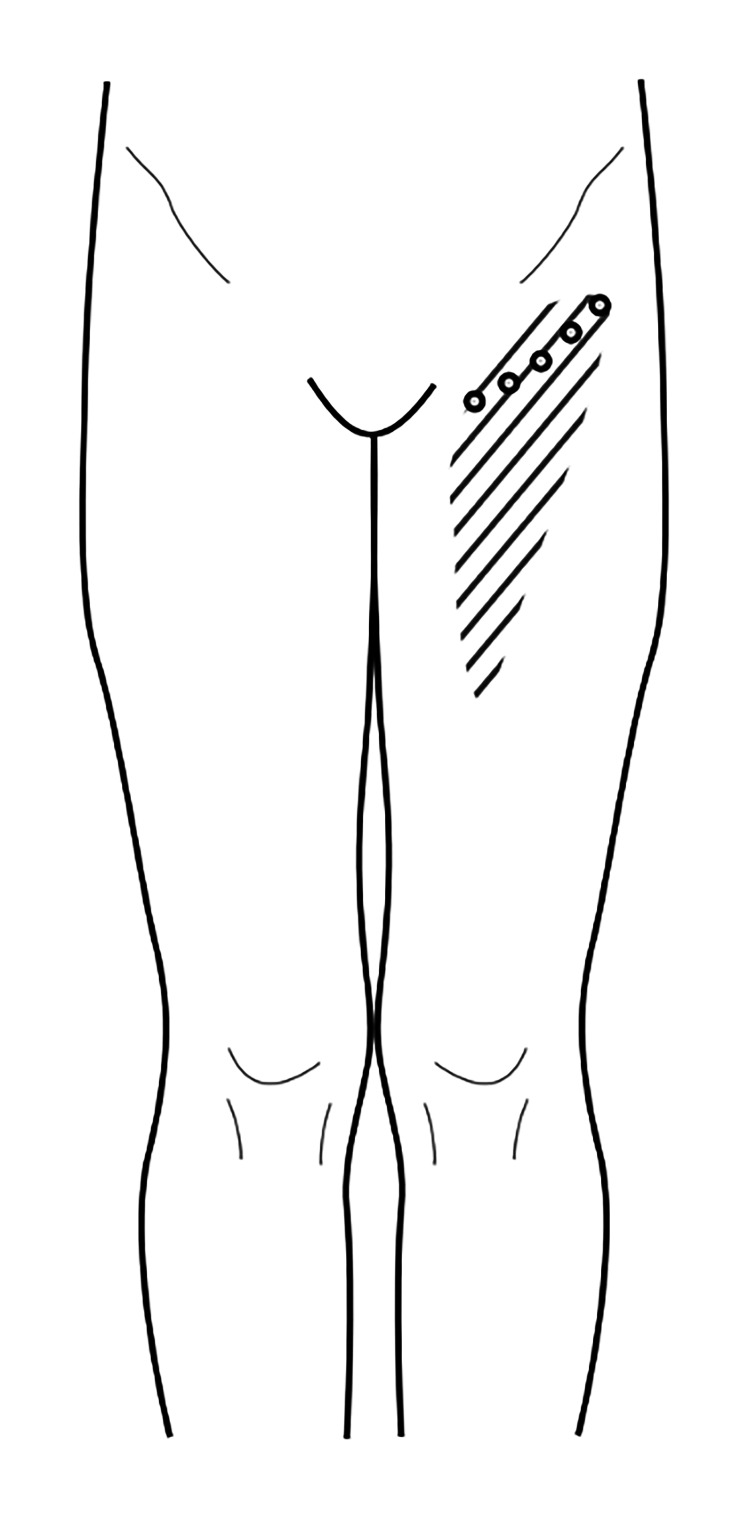
Glucopuncture injection targeting the superficial fascia distal to the inguinal ligament for left groin pain management This figure illustrates the application of glucopuncture injections administered to the superficial fascia located distal to the inguinal ligament, aimed at alleviating pain in the left groin area. The shaded region indicates the area affected by pain, while the marked dots signify the specific sites of glucopuncture injection. Image credit: Dr Kersschot.

During the clinical examination, the patient pointed to a pain region located below the inguinal ligament (as shown in the shaded area in Figure 1). Layered palpation of this area revealed several pain points within the superficial fascia caudal to the inguinal ligament. However, no muscular trigger points or pain points were found in the ipsilateral adductor muscle.

The patient demonstrated full ROM in both hips, and movement of either hip joint did not reproduce or exacerbate the groin pain. Interestingly, movement of the left hip joint provided partial, but not complete, relief of the groin discomfort. Examination of the lower back was unremarkable and did not indicate any abnormalities.

Based on the clinical findings, the patient's pain presentation did not align with degeneration of the hip joint or referral pain from other areas of the body. Because of the phobia of steroid injection, the patient opted to forgo further investigation at this time.

**Figure 2:**
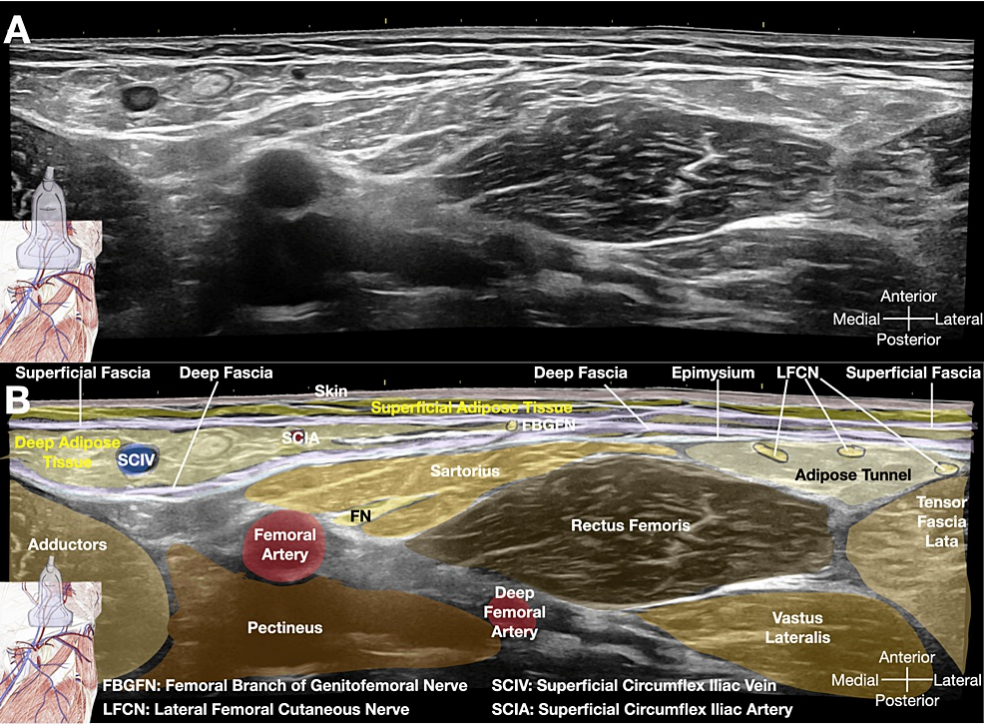
Sonoanatomy of the structures caudal to the inguinal ligament corresponding to the five dots in Figure 1 This figure demonstrates the sonoanatomy of the structures located caudal (inferior) to the inguinal ligament, corresponding to the five dots shown in Figure 1. Sub-figure A presents the original ultrasound image, while Sub-figure B provides a color-coded mapping of the structures, along with detailed labels for the anatomical features visible in Sub-figure A. Image credit: Dr. Lam K

**Figure 3:**
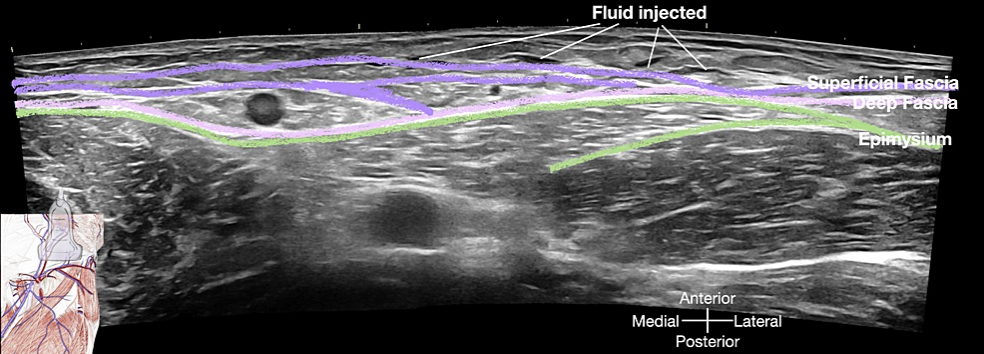
Ultrasound image of structures after palpation-guided injection (case 1) This ultrasound image depicts the anatomical structures in the target region immediately following the palpation-guided injection procedure for Case 1. Image credit: Dr. Lam K

He underwent a series of five injections into the superficial fascia using a 27-gauge, 1.5-inch needle (refer to Figure 1 and Video 1), with each session involving a total of 5 mL (5 x 1 mL) of 5% dextrose in water (D5W). Figure 2 was included to depict the sonoanatomy of the groin region below the inguinal ligament prior to the injection, highlighting the various layers of the myofascial system. Following the initial session, the pain subsided for a duration of 24 hours. After completing four consecutive weekly sessions, the pain completely resolved. Additionally, we deliberately performed an ultrasound scan of the injected regions, guided by palpation, to visually demonstrate the distribution of the injectate immediately after the procedure (Figure 3), as depicted in Video 1.

**Video 1:** Palpation/landmark-guided glucopuncture for chronic groin pain This video showcases a palpation/landmark-guided glucopuncture technique for managing chronic groin pain. The footage provides a clear illustration of the tangential needle approach employed in this procedure.

The shaded area in Figure 1 denotes the painful region of the patient. Our treatment aims to address this area by injecting the superficial fascia as shown by the five dots. The superficial fascia to the dermis of this shaded region is likely supplied by the anastomosis of the femoral nerve, the femoral branch of the genitofemoral nerve, and potentially some branches from the lateral femoral cutaneous nerve. The purpose of this manuscript is to describe a novel treatment using palpation-guided glucopuncture, which may alleviate chronic pain by delivering 5% dextrose tangentially through the needle puncture to the superficial fascia (Video 1). This approach offers a minimally invasive and potentially effective option for managing chronic pain in this region.

Clinical case 2

A 36-year-old woman experienced chronic pain in her left arm and neck for two years. The pain worsened when she held her arm above her head. She couldn't recall the exact onset of the pain but believed it was related to her job as a hairdresser. The pain was severe, reaching 7/10 on the NRS during the day and 8/10 at night, which often disrupted her sleep. She indicated that the pain typically started in the neck and radiated to the lateral part of the arm (shaded area in Figure 2). Despite trying various conservative treatments, including physiotherapy and occupational therapy, her pain persisted. NSAIDs prescribed by her doctor offered no relief and caused severe stomach pain, forcing her to discontinue their use. Normal MRI scans of the neck and ultrasound of the shoulder led her family physician to attribute the pain to stress and anxiety. Lorazepam was prescribed but proved ineffective.

**Figure 4:**
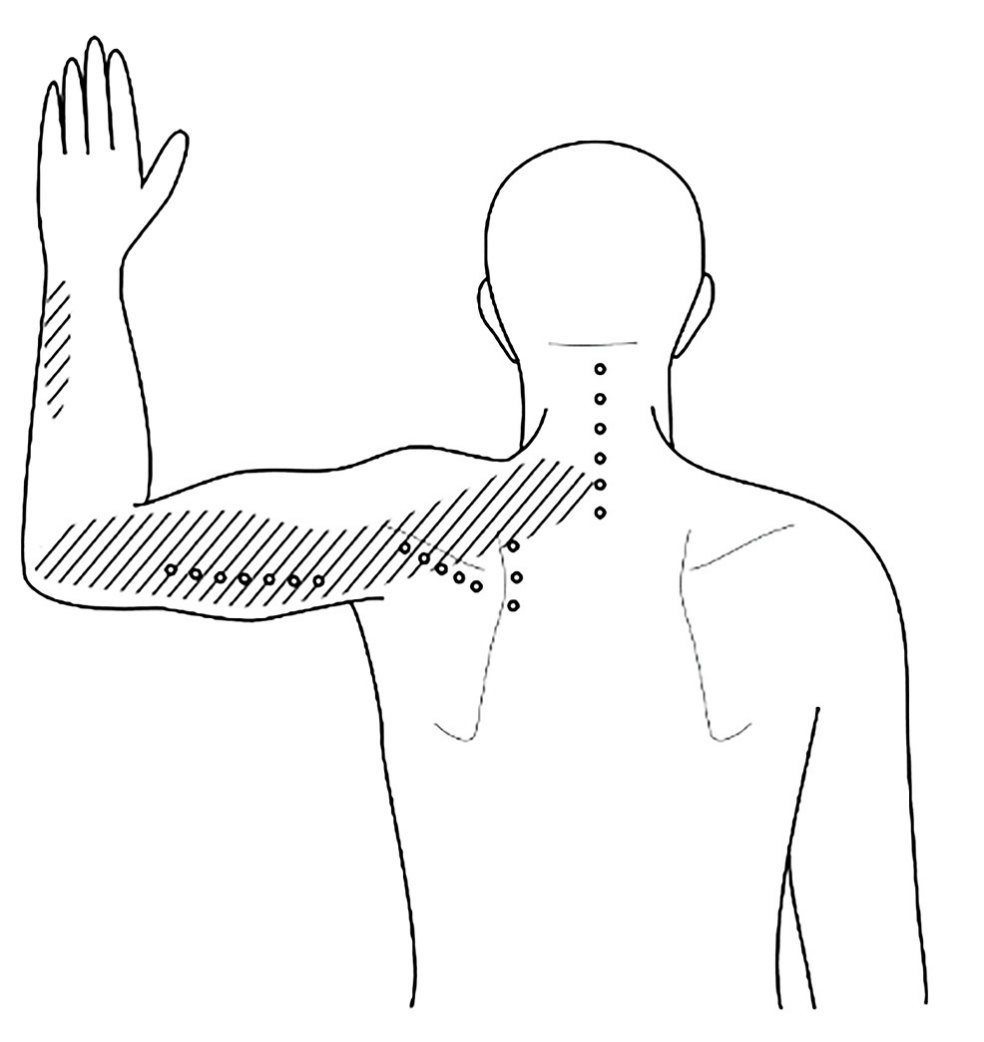
Glucopuncture injection strategy for treating the left arm and neck pain This figure demonstrates the glucopuncture injection approach targeting the neck, the medial border of the scapula, the vicinity of the scapula, and the lateral aspect of the triceps muscle to address pain in the left arm and neck. The shaded areas illustrate the regions affected by pain, while the dots indicate the precise locations of the glucopuncture injections. Image credit: Dr Kersschot.

Clinical examination revealed normal neuromuscular function but further examination revealed several painful areas upon light palpation (Figure 2). To address the patient's chronic pain, she received multiple injections of D5W into the fascia using a 27G, 2-cm needle. She received six injections in the neck, three at the medial border of the scapula, five near the spine of the scapula, and seven in the lateral part of the triceps muscle (as shown in the dots in Figure 4). Given her two-year history of chronic pain and very sensitive pain pattern, she received a total of 10 mL of D5W per session, with approximately 0.5 mL injected into each spot to minimize discomfort. The patient was informed about the possibility of a "reaction phase" (which might involve temporary worsening of pain and might be a sign of the body's self-repair mechanisms) after the injections. Following the first session, the pain intensified for approximately 48 hours before gradually subsiding spontaneously. After three sessions, the pain in both the neck and arm significantly improved, and night-time pain completely disappeared. After six weekly sessions, her pain had completely resolved, and the painful area in the lower arm vanished without requiring injections in that specific region.

Clinical case 3

**Figure 5:**
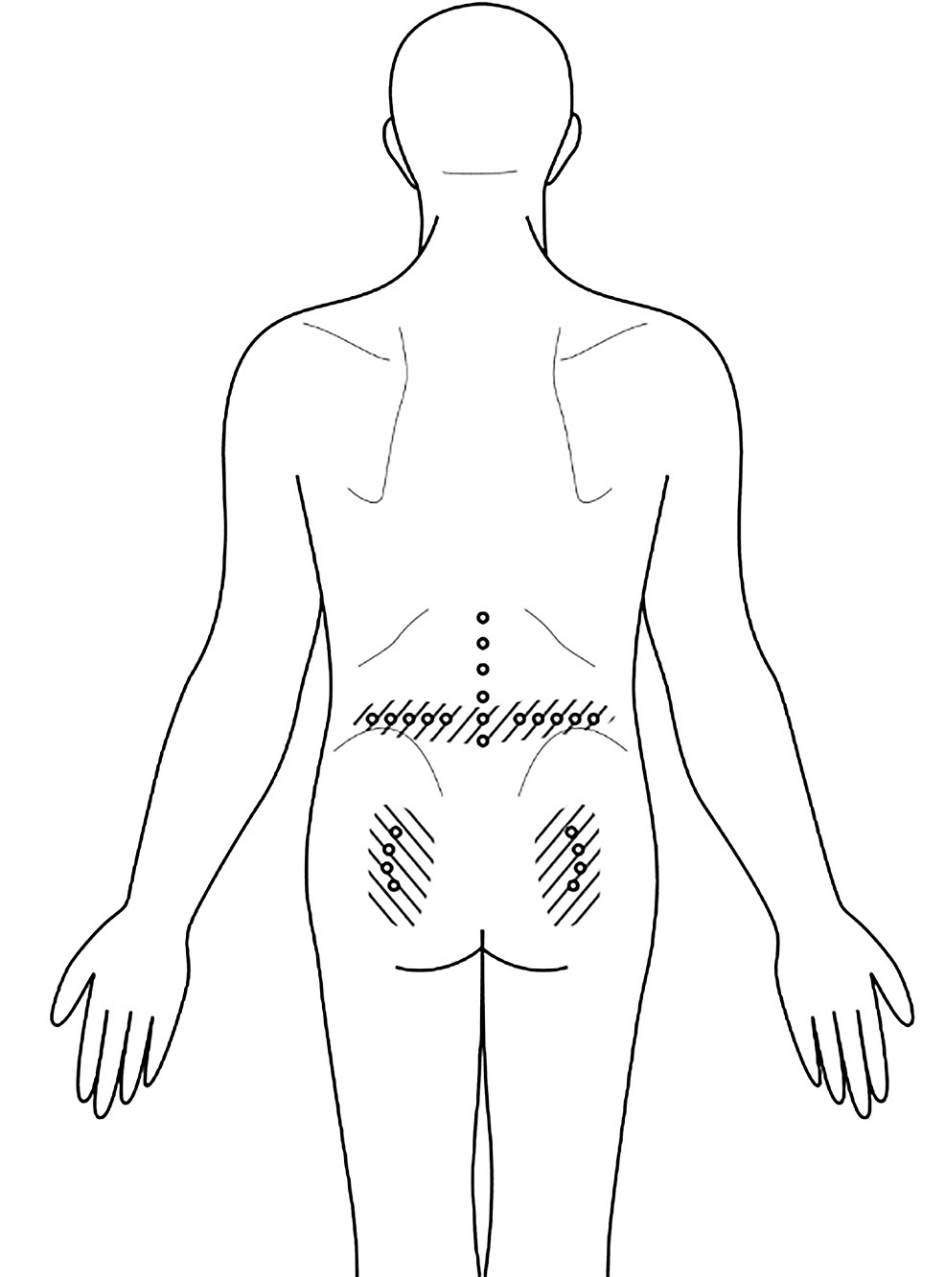
Fascial glucopuncture injection technique for low back pain relief This figure displays the application of fascial glucopuncture injections administered to the midline fascia of the low back, as well as to the left and right lumbar regions and the left and right gluteal areas, aimed at mitigating low back pain. The regions experiencing pain are depicted by the shaded areas, while the specific sites targeted by the glucopuncture injections are marked with dots. Image credit: Dr Kersschot.

A 67-year-old retired woman experienced low back and bilateral buttocks pain for six months. She described the pain as a dull, stabbing, and heavy sensation, rated 5-6 out of 10 on the NRS. There were no obvious causes for the pain, but she noticed it worsened when bending over, such as during or after gardening, or while sitting for long periods. The pain affected her hobbies, including gardening and reading, but fortunately did not disturb her sleep. When asked about the pain location, the patient indicated with her hands that the pain was present as a band in her low back (left, middle, and right side) and in both buttocks, as shown in the shaded area in Figure 5. 

The neurological and musculoskeletal examination of the lower back and hips were normal, except for some stiffness in the lower back and bilateral hips on active movement.

X-rays of both hips revealed bilateral hip degeneration, although she maintained a full range of passive movement and did not experience pain in hip movement. An MRI of the lumbar spine showed a disc hernia at L4-L5 on the right side, but the patient did not exhibit a typical pain pattern on the right back or leg. 

Having failed all previous conservative treatments, including pharmacological therapy with analgesics and anti-inflammatory medications, as well as non-pharmacological treatment with physiotherapy, she opted for a course of palpation-guided glucopuncture. She received multiple injections of D5W with a short 27G needle, totaling 10 mL per session. The injections were administered in the following areas (as shown in the dots in Figure 5): six in the low back (the pain points located within the superficial thoracolumbar fascia in the midline of the lower back), five in the left and right lumbar pain regions, and four in the left and right pain regions of the buttocks. After the first session, the patient experienced complete relief from low back pain for 24 hours, although the pain in her thighs remained unchanged. Following four weekly sessions, the patient's pain in her low back and thighs had completely resolved, and her yoga instructor noticed an improvement in her ROM in the low back and hips during class.

## Discussion

Fascial biotensegrity

Over the last decade, the FS has been widely accepted as a crucial and dynamic link between various systems in the human body. It is now regarded as an interconnected network from the head to the toes [1]. Because the FS is a complex network of membranes that extend throughout the body, numerous muscular expansions maintain it in a basal state of regional tension [1,9]. As previously discussed, the FS connects muscles, bones, and tendons as one integral system [9]. The FS is sometimes described as “the third system,” along with bones and muscles. 

While the FS is a major and crucial part of the MSK system, its clinical relevance is often overlooked by physicians and surgeons. Biotensegrity is a mechanical model that takes into consideration the interconnectedness of the fascia in the entire body [9-11] Biotensegrity offers a conceptual understanding of the hierarchical organization of the human body and explains the body's adaptability to change [11,12]. The theoretical model may also help explain why injections with D5W in one region can influence other parts of the MSK system [12].

Superficial and deep fascia

The FS links skeletal muscles and bones by forming a body-wide network of multidirectional myofascial continuity [12]. The FS itself consists of two sheets of connective tissue. One is found below the skin, which is referred to as the superficial fascia, and the second lies deeper in the body, referred to as the deep fascia [12,13].

The superficial fascia 

**Figure 6:**
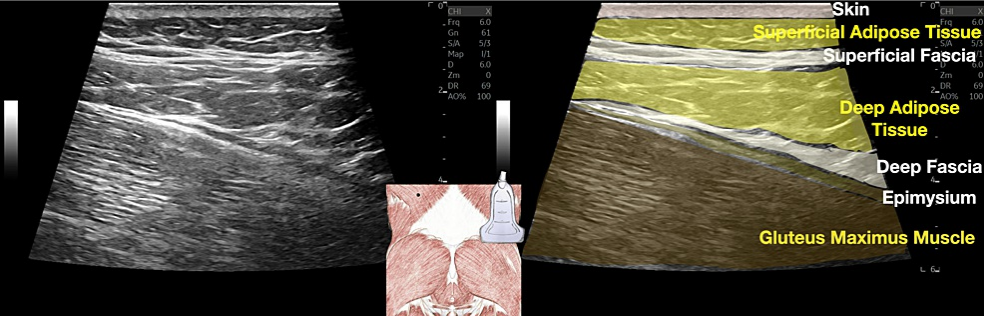
Ultrasound imaging of fascial layers of the gluteal region This figure provides a detailed illustration of the relationships between the skin, superficial adipose tissue, superficial fascia, deep adipose tissue, deep fascia, epimysium and the underlying musculature of the gluteus maximus. The image on the left presents a B-mode ultrasound representation, while the image on the right offers a color-coded mapping of the various layers, accompanied by their corresponding anatomical labels. This comprehensive ultrasound representation enables a detailed understanding of the layered arrangement of the soft tissues in the gluteal region. The ability to clearly delineate the superficial and deep fascial planes, as well as their relationship to the surrounding adipose and muscle tissues, is crucial for accurately targeting these structures during interventional procedures. Image credit: Dr. Lam K

The superficial fascia is the fibrous layer localized between the superficial adipose tissue and deep adipose tissue (Figure 6), and its layers are important to maintain regional ROM. The superficial fascia forms a network that acts as a subcutaneous wetsuit connecting different areas of the body [14]. An injured superficial fascia can lead to both regional pain and pain at a distance because of its nature as an underground network of interconnecting bands of connective tissue. 

The shaded areas in Figures 1, 4, 5 correspond to the pain regions or zones described by the patients. In this case series, these pain areas are postulated to originate from the superficial fasciae. It is important to note that pain associated with superficial fasciae defects is typically superficial, vague, and poorly defined. These anomalies or defects in the superficial fascia often manifest as local pain or refer pain to distant parts of the body. 

The deep fascia 

The deep fasciae also form an invisible network that connects and supports the deeper muscles of our body. The deep fasciae are also important for protection of fragile tissues such as nerves, ganglia, and blood vessels, for example, the prevertebral fascia and carotid sheath, which cover nerves, ganglia, and blood vessels. These deep fascial tissues attach, stabilize, and separate muscles, vessels, and organs in areas of the body such as the thorax, abdomen, and pelvis. One such example is the pleura, which envelops and protects the lungs, ensuring their proper functioning. Another example is the pericardium, which encloses and shields the heart, providing it with necessary support and insulation. Additionally, the endopelvic fascia is another type of deep fascia that deserves mention. It can be further categorized into a parietal sheet, which covers the pelvic wall medially, and a visceral sheet, which aids in suspending and stabilizing the viscera within the pelvic cavity. These deep fasciae collectively contribute to the structural integrity and functionality of the body's vital organs. Deep fascial components also exhibit dense innervation, encompassing many nociceptive afferents, which means that these deep fasciae are also important for pain management [13]. In other words, patients may complain about vague pain in the thorax, abdomen, or pelvic area, which primarily originates from the deep fascia and may remain invisible through ultrasound, MRI, or CT. 

Connection between superficial and deep fascia

The connection between the superficial and deep fasciae is primarily through a zone of transition, where the two layers blend together. This zone is characterized by a gradual increase in density and thickness of the fascial tissue, as you move from the superficial to the deep layer. At this interface, the superficial fascia sends septal extensions into the deep fascia, which helps to anchor the two layers together. These septal extensions also create compartments that separate the superficial adipose tissue from the deep adipose tissue, allowing for distinct functional and anatomical regions.

The connection between the superficial and deep fasciae is not only structural but also functional. The two layers work together to facilitate movement, maintain posture, and regulate proprioception (our sense of body position and movement). For example, during muscle contraction, the deep fascia helps to transmit forces to the superficial fascia, which then distributes these forces across the skin and underlying tissues. As both systems contain multiple nociceptors, they act as a second sensory system that operates parallel to peripheral nerves. More research in this field is required to confirm this hypothesis. 

In the context of ultrasound-guided fascial hydrorelease/hydrodissection, understanding the connection between the superficial and deep fasciae is essential for accurate targeting and treatment of the FS. By recognizing the relationships between these layers, clinicians can better appreciate the complex anatomy of the FS and develop more effective treatment strategies.

MSK pain and dysfunction related to fascia: two major mechanisms

The FS is both a biomechanical construction as well as a large sensory organ containing many sensory receptors. Thus, management of the fascia can regulate both biomechanical dysfunction and regional pain. Damage to the fascia can cause significant performance deficits in high-performance sports and recreational exercise, but it also contributes to the development of MSK disorders and persistent pain in the elderly [5]. As mentioned previously, the FS can lead to acute and chronic MSK complaints through two major mechanisms, mechanical and sensory dysfunction. 

Tightness of the fascia and restricted ROM

The intimate connection of fascia with muscle provides the opportunity for immediate force transmission. Fascia has elasticity which allows it to both retain its shape and respond to deformation. Fascial tissue can become tight or restricted due to poor posture, repetitive strain, regional inflammation, acute overuse, or even stress. Such fascial lesions are often quite small but can also span over larger areas. Such fascial restrictions may lead to reduced ROM, such as restriction of shoulder movement, reduced neck rotation, or restriction of the back in bending forward while standing. Currently, doctors and therapists mainly focus on the superficial layer because it is easy to inject and treat manually. In particular, physical therapists and osteopaths have been treating fascia for several decades. Multiple techniques in the field of physiotherapy make the superficial fascia less tight or restricted, such as mobilization, stretching, and specific massage techniques. These manual therapies aim to restore flexibility, improve movement, and reduce discomfort. In this article, we focus on the treatment of the superficial fascia with glucose 5% (or dextrose 5%) injections. 

Fascial nociceptors: an overlooked source of pain? 

The FS is not just an elastic packaging system for muscles and organs but also a richly innervated system [12]. Fascial tissue contains a large number of proprioceptors and nociceptors, much more than muscle tissue [13]. Proprioceptors signal the positioning of the body and are important in everyday life, especially during sports activities. The nociceptors provide warnings of the presence and location of injury or inflammation in or near the fascia. These nociceptors become irritated when overused and in cases of joint degeneration or tendinopathy. In addition, underlying bursitis or joint arthritis may also irritate the superficial fascia. Collectively, fascial nociceptors may be an important source of regional pain syndromes. 

The FS has been described as the largest sensory organ, containing 250 million nerve endings [12]. However, most doctors and physical therapists are hardly aware of its magnitude. Nerve endings that have been identified in the FS include Ruffinian bodies in the aponeurosis, Pacinian bodies in the visceral fascia, Golgi organs in tendons and ligaments, interstitial nerves, free nerve endings, and C-fibers [15]. The abundance of nociceptors in fascial tissues may explain the importance of the FS in the management of regional MSK pain of unclear origin [5]. Fascial injuries can be crucial in regional pain syndromes which span different muscles, tendons, ligaments, or joints. Patients who complain of vague pain regions typically visit orthopedic surgeons without a well-defined diagnosis. Most physicians then aim to seek an “objective sign” on MRI or ultrasound that may explain the complaints. That objective sign may then be used as a stepping stone to treat vague pain through means such as steroid injections or surgery. We postulate here that the FS may be an invisible but crucial element in these patients. More research is required to investigate the extent to which the FS plays a role in the development of vague MSK pain syndromes. Ultrasound equipment with better resolution may bring more light to this undervalued topic as technology progresses in the near future.

Treatment of MSK pain with glucopuncture into the regional fascia 

Glucopuncture is a term introduced to describe regional injections with low concentrations of D5W into the dermis, fascia, muscles, and ligaments [16]. Injections can also be applied perineurally [17], into joint cavities, or in the epidural space. Typical injectates are glucose 5% in water or D5W. Over the last decade, D5W injections have become increasingly popular worldwide, although research in this field remains limited. Both palpation-guide glucopuncture and ultrasound-guided glucopuncture have been applied [16,17].

Glucopuncture has two approaches: palpation/landmark-guided and ultrasound-guided. While ultrasound-guided glucopuncture is more accurate and necessary for delivering glucose to perineural structures, deep joints, and structures for safety reasons, palpation-guided glucopuncture is especially suitable for treating superficial fascia, ligaments, muscles, and tendons. This is particularly relevant in low-income settings where ultrasound may not be readily available.

This manuscript focuses on the palpation/landmark-guided injection technique for treating the superficial fascia and superficial adipose tissue. The needle is typically directed tangentially to the skin, targeting the superficial fascia and/or superficial adipose tissue located only a few millimeters to 1 cm below the dermis. During the procedure, puncturing of visible superficial arteries and veins is deliberately avoided.

It is important to note that for palpation-guided glucopuncture to treat ligaments, muscles, and tendons, a more perpendicular and deeper needle placement, typically 2-4 cm for most of the ligaments, muscles and tendons (unless we are dealing with trigger points of gluteal muscles which can be as deep as 8cm), would be adopted compared to the approach described in this manuscript.

When performing palpation/landmark-guided glucopuncture for superficial fascia in this groin area, as described in Case 1, even though the femoral bundle, a group of major nerves and vessels located in the thigh, lies deeper than the target tissues (as illustrated in Figures 2, 3), it is still crucial to pay particular attention about the needle direction, especially in those very thin patients. Proper anatomical knowledge and careful needle placement are always essential. 

In our protocol for palpation-guided glucopuncture to treat chronic pain associated with superficial fascia pathologies, we typically inject 0.5 to 1 ml of 5% dextrose/glucose per injection site. The specific volume used for each spot depends on several factors, including (i) Patient sensitivity and tolerance: More sensitive patients may require lower volumes to minimize discomfort; (ii) Chronicity of pain: Patients with more chronic fascial pain may have increased inflammation and sensitivity, making them more susceptible to "reaction pain" - exacerbated pain in the first to second night after injection. Our experience suggests that reaction pain is more likely in chronic fascial pain patients following palpation-guided glucopuncture; (iii) Location of injection: To avoid triggering severe reaction pain, we avoid injecting directly into pain points and instead inject around them. Additionally, we avoid injecting into visible superficial arteries and veins.

By considering these factors, we can tailor the injection volume to each patient's individual needs and minimize the risk of adverse reactions. The volume of injectate administered at each injection site varied between the different cases in this study. This variability was intentionally incorporated to account for individual anatomical differences and optimize the therapeutic delivery. This tailored approach to injectate volume allowed the clinicians to precisely distribute the D5W within the relevant myofascial structures for each individual patient, optimizing the therapeutic efficacy while minimizing the risk of complications.

Variability in patient response is to be expected, similar to cases involving steroid injections. The clinical experience we are sharing here spans several decades across multiple physicians. Determining which patients are more likely to experience a reaction phase or exacerbated pain will require further large-scale, randomized controlled trials to draw definitive conclusions. The complexity of chronic pain conditions and individual patient factors make predicting the response to treatment challenging.

Continued research and careful monitoring of patients undergoing this novel palpation-guided glucopuncture approach will help refine the understanding of which patients are most likely to benefit and how to best manage any temporary worsening of symptoms. Maintaining an open dialogue with patients about the potential for a reaction phase is important to set realistic expectations and ensure appropriate follow-up care.

Maintaining sterility and potential side effects of glucopuncture

Sterility Precautions

To ensure the safety of the procedure, strict sterility protocols are followed at all times. This includes measures such as wiping the skin with antiseptic solution and using a sterile needle and syringe for each injection site. This helps prevent the introduction of pathogens, such as Staphylococcus, and reduces the risk of infection.

Single Needle Usage

While the ideal approach would be to use a new syringe and needle for each injection site, this may not always be practical. In such cases, the same needle can be used for multiple injections, similar to the technique employed by surgeons when closing a wound. However, the clinicians remain vigilant in maintaining aseptic techniques to minimize the risk of cross-contamination.

Potential Side Effects

The available data on the side effects of palpation-guided glucopuncture is limited, as this is a relatively new technique. However, based on clinical experience, the following observations can be made:

Allergy to dextrose or glucose: This is a rare occurrence, estimated to affect approximately 2 in 10,000 injections, especially if the patient is allergic to corn as most glucose solution is derived from corn. 

Increased pain after the procedure: This is a relatively common occurrence, affecting around 1 in 10 patients. However, the increased pain usually subsides within 1-2 days.

Bruising: Localized bruising or blue spots may occur in around 1 in 2 cases, but these typically resolve within 1-2 days.

Persistent pain beyond two days: Serious pain that lasts longer than two days after the injection is uncommon, occurring in approximately 1 in 1,000 cases. This rate is lower than that observed with steroid injections for the same indication, although further studies are needed to confirm the comparative safety.

It is important to note that the potential side effects of palpation-guided glucopuncture appear to be similar to those observed with steroid injections, but potentially less severe due to the use of a thinner needle and the relatively harmless nature of the injectate (D5W). Additionally, the risk of certain complications, such as bruising, may be higher when multiple injections are performed.

For deep fascial injections, the use of ultrasound guidance is mandatory to ensure accurate targeting and minimize the risk of complications. In these cases, a single injection per session is typically recommended.

Clinical application of glucopuncture for fascial pain and dysfunction

The clinical relevance of fascial mobility and myofascial pain is largely understudied, but a better understanding of these phenomena could result in improved care for patients with vague MSK pain that cannot be explained by specific signs on X-rays or MRI. Physicians who treat patients with MSK pain or sports injuries on a daily basis often observe that patients complain about pain patterns that do not follow a strict medical diagnosis. Some patients, for example, may show with the left hand that their pain region spans from the neck to the right scapula and triceps region, while X-ray and ultrasound of the neck and shoulder are normal. Some may complain about symmetrical pain patterns in the left and right shoulders, while imaging shows calcific tendinopathy on the left side only. Other patients may complain about pain in and around the knee, radiating to the patellar tendon and tibial tuberosity, while no objective signs of knee joint arthritis, meniscal tears, or prepatellar bursitis can be identified. Additionally, some patients may experience pain in the low back without any signs on MRI of facet joint degeneration, while others experience pain in the low back and hip, radiating to the right knee, which does not correspond to the typical referral pattern of a herniated disc or facet joint degeneration. Patients may have chronic groin pain, while MRI of the hip joint is normal, or may complain about low back pain radiating into the knee and ankle, which does not correspond to a trigger point in the sacroiliac ligament nor a trigger point in the gluteus minimus muscle. Some patients also complain about pain in the neck and both trapezius muscles without any signs on CT or MRI of a cervical disc hernia or facet joint degeneration. These patients may leave even experienced orthopedic surgeons with a sense of frustration because the conventional diagnostic tools do not match the clinical picture. Sometimes, these complaints are due to mental disturbances or lack of movement, but we postulate here that the FS may play a crucial role in these patients. 

The clinical experience illustrates that injecting the FS with low concentrations of sugar water leads to local connective tissue repair and may lead to less tightness in the fascia. This may contribute to a regional biomechanical release in the MSK system.

Glucopuncture may also influence the nociceptors in the fascia itself, leading to pain modulation in larger regions. In some cases, both regional injections and distal injections into the superficial fascia have been applied. Randomized controlled trials should be designed and conducted to support our clinical findings.

Remarks

Most injections into the FS are easy and safe to apply because they are injected into the superficial layer. However, the deep layers are equally important and require ultrasound guidance to inject the fascia in deeper musculoskeletal structures of the thorax, abdomen, or pelvic area.

Ultrasound-guided fascia hydrorelease (US-FHR)

US-FHR or ultrasound-guided fascia hydrodissection (US-FHD) have recently gained popularity as therapeutic options for the management of fascial pain [18-20]. The term "hydrorelease" and "hydrodissection" refers to the separation and relaxation of fascial structures using the hydraulic force of injected solutions, typically D5W (5% dextrose in water). In the case presented in Video 2, we demonstrated the application of US-FHR/US-FHD for the treatment of chronic groin pain. The video clearly illustrates the needling approach under ultrasound guidance, targeting the superficial fascia in the affected region.

**Video 2:** Ultrasound-guided hydrorelease/hydrodissection of superficial fascia for chronic groin pain This video presents a minimally invasive approach for managing chronic groin pain through ultrasound-guided hydrorelease/hydrodissection of the superficial fascia. This technique involves injecting a solution, typically D5W (5% dextrose in water), into the superficial fascia under real-time ultrasound guidance. The injected solution separates and relaxes the fascial layers, potentially alleviating pain and improving function. Ultrasound guidance allows for precise targeting of the affected fascia, minimizing the risk of complications and ensuring accurate delivery of the therapeutic solution. This technique has shown promise in treating various pain syndromes, including chronic groin pain, frozen shoulder, and low back pain. While further research is needed, existing studies suggest that ultrasound-guided hydrorelease/hydrodissection can be an effective and safe treatment option for chronic pain.

**Figure 7:**
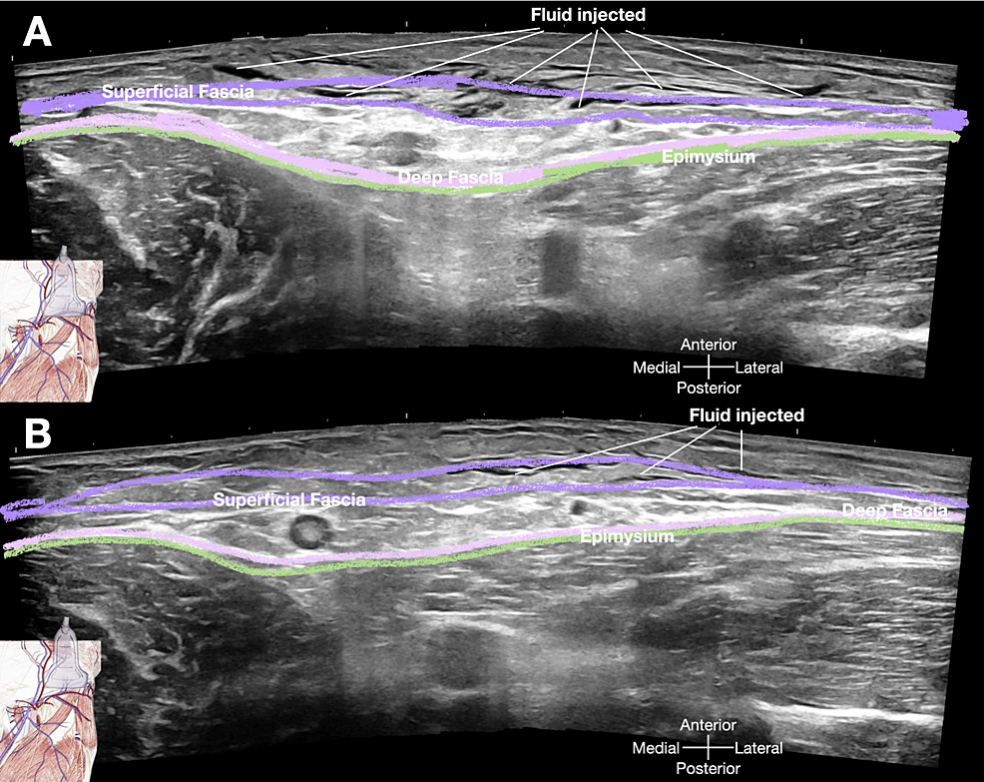
Post-ultrasound-guided hydrorelease/hydrodissection ultrasound findings A: This image, captured 30 seconds after the injection, demonstrates the immediate dispersal of the injected solution within the superficial fascia and surrounding soft tissues. The solution appears as a diffuse, hypoechoic/ anechoic area within the previously hyperechoic fascial plane, indicating successful separation and relaxation of the fascial layers. Additionally, the underlying structures, such as the adductor muscle group and the femoral neurovascular bundle, are clearly visualized, confirming the absence of inadvertent spread of the solution to these critical areas. B: This image, obtained two minutes post-injection, shows further spreading of the injectate. The fascial plane appears less echogenic compared to the pre-injection state. Image credit: Dr. Lam K

The immediate post-injection ultrasound findings, captured at 30 seconds and 2 minutes, are presented in Figures 7A, 7B respectively.

These images depict the diffuse distribution of the injected solution within the superficial fascia and surrounding soft tissues, as well as the delineation of the underlying adductor muscle group and the femoral neurovascular bundle. 

The utilization of ultrasound guidance throughout the procedure allowed for precise targeting of the affected fascial structures and real-time monitoring of the injectate dispersal. This technique has also been reported to be effective in the management of pain syndromes in the shoulder, low back, and post-arthroscopic knee scarring [18,19].

While not the primary focus of this article, the application of ultrasound-guided deep fascia injections is an emerging and fascinating subject in the field of pain management. 

## Conclusions

In the past decade, the global medical community has recognized the fascial system's importance in MSK pain syndromes due to its nociceptor-rich nature. Glucopuncture has emerged as a potential pain modulation technique for patients with musculoskeletal pain without clear imaging abnormalities. Caution is advised in interpreting the efficacy of glucopuncture without robust scientific backing to prevent patient misinterpretation. Collaboration and well-designed trials are crucial to validate glucopuncture's efficacy and safety globally. Palpation-guided injections in glucopuncture offer a practical intervention, especially in resource-limited healthcare settings, aiming to advance its therapeutic potential for MSK pain.
